# Machine learning assists prediction of genes responsible for plant specialized metabolite biosynthesis by integrating multi-omics data

**DOI:** 10.1186/s12864-024-10258-6

**Published:** 2024-04-29

**Authors:** Wenhui Bai, Cheng Li, Wei Li, Hai Wang, Xiaohong Han, Peipei Wang, Li Wang

**Affiliations:** 1 College of Computer Science and Technology (College of Data Science), Taiyuan University of Technology, Taiyuan, 030024 China; 2 Shenzhen Branch, Guangdong Laboratory of Lingnan Modern Agriculture, Key Laboratory of Synthetic Biology, Ministry of Agriculture and Rural Affairs, Agricultural Genomics Institute at Shenzhen, Chinese Academy of Agricultural Sciences, China 518000 Shenzhen,; 3 Kunpeng Institute of Modern Agriculture at Foshan, Agricultural Genomics Institute at Shenzhen, Chinese Academy of Agricultural Sciences, Shenzhen, 518124 China; 4 National Maize Improvement Center, Key Laboratory of Crop Heterosis and Utilization, Joint Laboratory for International Cooperation in Crop Molecular Breeding, China Agricultural University, Beijing, 100193 China

**Keywords:** Machine learning, Plant specialized metabolites, Genomics, Proteomics, AutoGluon-Tabular

## Abstract

**Background:**

Plant specialized (or secondary) metabolites (PSM), also known as phytochemicals, natural products, or plant constituents, play essential roles in interactions between plants and environment. Although many research efforts have focused on discovering novel metabolites and their biosynthetic genes, the resolution of metabolic pathways and identified biosynthetic genes was limited by rudimentary analysis approaches and enormous number of candidate genes.

**Results:**

Here we integrated state-of-the-art automated machine learning (ML) frame AutoGluon-Tabular and multi-omics data from *Arabidopsis* to predict genes encoding enzymes involved in biosynthesis of plant specialized metabolite (PSM), focusing on the three main PSM categories: terpenoids, alkaloids, and phenolics. We found that the related features of genomics and proteomics were the top two crucial categories of features contributing to the model performance. Using only these key features, we built a new model in *Arabidopsis*, which performed better than models built with more features including those related with transcriptomics and epigenomics. Finally, the built models were validated in maize and tomato, and models tested for maize and trained with data from two other species exhibited either equivalent or superior performance to intraspecies predictions.

**Conclusions:**

Our external validation results in grape and poppy on the one hand implied the applicability of our model to the other species, and on the other hand showed enormous potential to improve the prediction of enzymes synthesizing PSM with the inclusion of valid data from a wider range of species.

**Supplementary Information:**

The online version contains supplementary material available at 10.1186/s12864-024-10258-6.

## Background

Plants synthesize a huge number of specialized metabolites that allow them to interact and adapt to the ever-changing environment. There are more than 200,000 PSM estimated, with vast specificity in different species, tissues or after different stimulations [1]. Some of these compounds are high-value nutraceuticals and pharmaceuticals, which has primed a desire to delineate their biosynthetic genes and pathways in various organisms [2–4]. Based on their biosynthetic pathways, specialized metabolites are usually classified into several molecule families, among which three have the largest number: terpenoids, alkaloids and phenolics [5, 6]. For example, essential oils (mainly comprising monoterpenes) are used for aromatherapy and medicine [7–9], while caffeine, the most well-known alkaloid [10], and salicylic acid, the active ingredient in aspirin, have established medical applications [11]. Hence, the discovery of various specialized metabolites and their biosynthetic pathways is likely to have a significant impact on our production and life [12–14].

However, uncovering new genes that synthesize specialized metabolites is challenging due to the tremendous complexity of metabolic networks and the absence of standard chemicals for intermediate products [15, 16]. Despite the numerous challenges, we have accumulated substantial knowledge of metabolic pathway genes through biochemical and genetic approaches over the past few decades [13, 17, 18]. With the ever-increasing availability of multi-omics data, candidate genes responsible for primary and specialized metabolism have been predicted computationally. For example, pathway memberships of genes could be identified based on co-expression profiles [19, 20], co-localization in cellular compartment [21], as well as biosynthetic gene clusters [22, 23]. Although these methods have produced notable results in various species or fields, the selection and functional validation of a massive number of candidate genes makes the discovery of metabolites and related genes time-consuming and expensive [24, 25]. Noteworthily, Moore et al. [26] used machine learning (ML) algorithms to classify primary and specialized metabolic genes from multiple characteristics with high prediction accuracy. However, the Moore et al. [26] did not explore genes participating in different specialized metabolic pathways. Later on, Wang et al. [20] used ML algorithms to predict genes in 85 metabolic pathways in tomato. However, the best model accuracy in this study was 58.3%, which means the prediction may generate misleading results when distinguishing genes in multiple individual metabolic pathways.

**Fig. 1:**
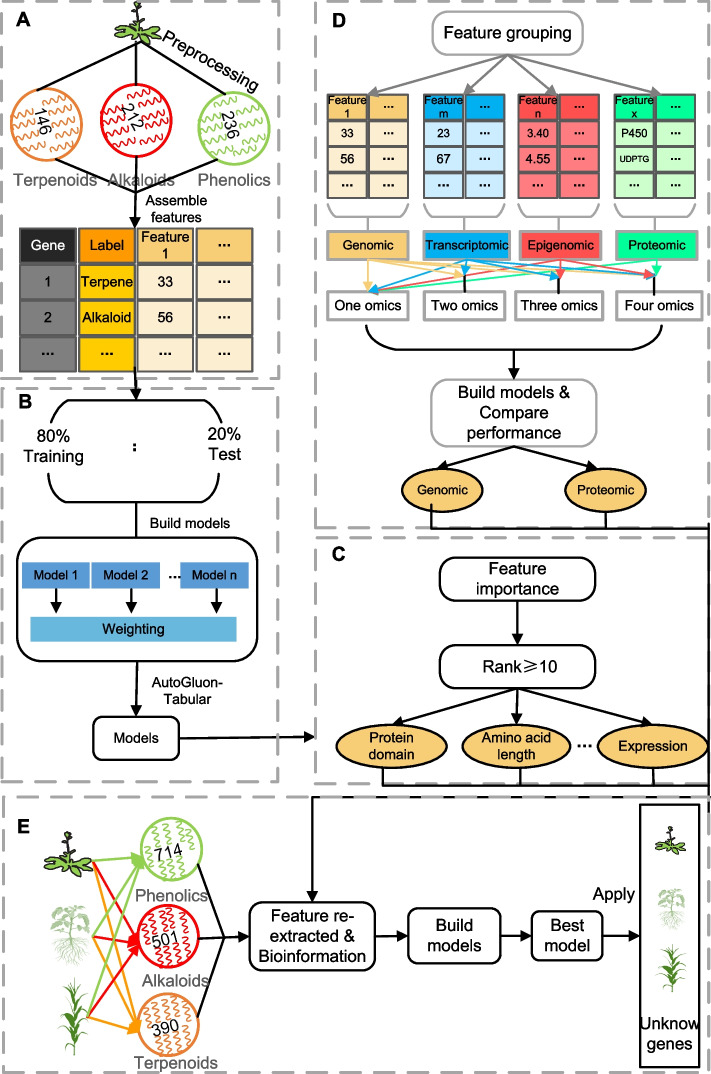
Diagram illustrating the research workflow. **A** Multi-omics features (data) from *Arabidopsis* collection and preprocessing. **B** The genes with properties (referred to as features) were split into training (80%) and test (20%) sets, and machine learning (ML) models were built and tuned to predict the enzymes synthesizing plant specialized metabolites (PSM). **C** Comparing importance of individual features separately. **D** Assessing importance in groups of omics features. **E** Essential features were picked from *Arabidopsis* and then re-extracted in *Arabidopsis*, maize and tomato to be used to create a three-species ML model to predict enzymes synthesizing PSM in these three species

Here we utilized multi-omics feature data from *Arabidopsis* to build ML models with state-of-the-art techniques (AutoGluon-Tabular) [27] for the prediction of genes synthesizing terpenoids, alkaloids, and phenolics (Fig. 1A, B), and assessed the essential features contributing to the model performance (Fig. 1C, D). Using these key features, we built a new model which performed better than the model built with all features. Furthermore, we constructed a three-species (*Arabidopsis*, tomato and maize) model using corresponding key features from these species (Fig. 1E), which rendered either equivalent or superior performance to intraspecies predictions. The external validation results in grape and poppy suggested our three-species model not only predicted enzymes synthesizing PSM with high accuracy, but could also be potentially extended to further improve the prediction of enzymes synthesizing PSM as data increases from other species.

## Results

### Datasets, features and machine learning algorithms

*Arabidopsis thaliana* has been thoroughly studied gnomically and genetically and has accumulated a sufficient amount of omics data, making it possible to build enzymes synthesizing PSM predicting models with high accuracy and assess the essential features linked to the models [26, 28]. Given that there are frequently less than 10 genes in a single SM pathway, we concentrated on a wide range of metabolic domains to train models effectively [20]. On the Metabolic Gene Cluster Viewer (https://metabolicclusterviewer.dpb.carnegiescience.edu/#!/cluster/v1/search), 363 alkaloids with the Metabolic Domains “Nitrogen-Containing Compounds”, 381 phenolics with the Metabolic Domains “Phenylpropanoid Derivatives”, and 207 terpenoids with the Metabolic Domains “Terpenes” were identified in *Arabidopsis* (Search parameters: Species, “Arabidopsis thaliana col (3702)”; Protein Feature (Metabolic Domains), “Nitrogen-Containing Compounds, Phenylpropanoid Derivatives, and Terpenoids”). Since we aimed to build models to distinguish genes from these three main metabolic categories, hereafter, these three Metabolic Domains are referred to as metabolic pathways in this paper. There are only 16 genes (1 gene involved in terpene and phenolic biosynthesis; 15 genes involved in alkaloid and phenolic biosynthesis) involved in multiple pathways after our strict selection. Since the ML algorithm we used only allows prediction of a gene to one class, and incorporating genes involved in multiple pathways would bewilder the algorithm, thus genes that were involved in multiple metabolic pathways were excluded beforehand.

Furthermore, based on the genes obtained above and their features employed by Moore et al., (2019), we obtained a total of 195 terpenoids, 354 alkaloids, and 363 phenolic genes involved in terpene, alkaloid, and phenolic biosynthesis respectively (Original dataset, Additional file 2: Data S1) with abundant features. There were 80 numeric features, and one text feature containing a string representation of the protein domains associated with each gene (see Methods). These numeric features inherited from Moore et al. (2019) were generally divided into three categories: features related to gene expression/co-expression, chromatin accessibility, and protein domain. As gene expression, especially co-expression, is often utilized to determine candidate genes in metabolic pathways based on the assumption that genes in the same pathway are co-expressed, and chromatin accessibility is believed to affect gene expression, we incorporate them into our feature analysis. And the three categories have 28, 9, 43 features, respectively. The feature of gene expression/co-expression contain the expression values (median, max, variation or breadth) of different developmental stages, the number of conditions (abiotic, biotic or hormone stimuli) under which a gene was up or down-regulated and the max Pearson correlation coefficients to paralog, specialized metabolism or general metabolism genes under different condition (developmental stages, abiotic, biotic or hormone stimuli). There are eight average values of histone marks and one CG methylation in chromatin accessibility. The numeric features related to protein domain is a binary table, showing pfam domains where 1 means the presence of the corresponding domain and 0 means the absence, which makes different protein domain as different features. The detailed description of features was summarized in Additional file 2: Data S2. Although only a few gene sequence-related features were utilized when predicting SM genes by Moore et al. (2019), sequence-related features provided important information in models predicting cold-responsive genes by Meng et al. (2021). Therefore, 120 sequence related features were obtained in *Arabidopsis* by referring to the method of Meng et al. (2021). We further narrowed down our datasets to include a smaller set of genes with PMID, which means the metabolic functions were supported by experimental validation in previous research (see Methods). This led to a gold standard (GS) dataset, which comprised 146, 212, and 236 genes involved in terpenoid alkaloid, and phenolic biosynthesis (Fig. 1A; Additional file 2: Data S3). The original and GS datasets were utilized to build ML models. While we initially focused on *Arabidopsis* due to the availability of extensive multi-omics data, the importance of model generalizability impels us to subsequently verify the model’s performance by incorporating gold standard datasets of tomato and maize.

An automated ML algorithm, AutoGluon-Tabular, was used to build predictive models [27]. This algorithm fits various “base” models including Random Forests (RF) [29], LightGBM boosted trees (LightGBM) [30], CatBoost boosted trees (CatBoost) [31], Extremely Randomized Trees (ExtraTrees) [32], XGboost [33] and neural networks (NeuralNetMXNet and NeuralNetFastAI), which are ultimately assembled in a linear way and the performance of the final integrating algorithm of AutoGluon-Tabular was evaluated using 11 tabular datasets chosen from Kaggle competitions [27]. In particular, these “base” models are individually trained with conventional pipelines. Subsequently, the final ensemble model is trained with the predictions of the base models as its features. Hyperparameter details and weights of the base models are provided in Additional file 1: Table S1.

### Model construction and evaluation in *Arabidopsis*

**Fig. 2:**
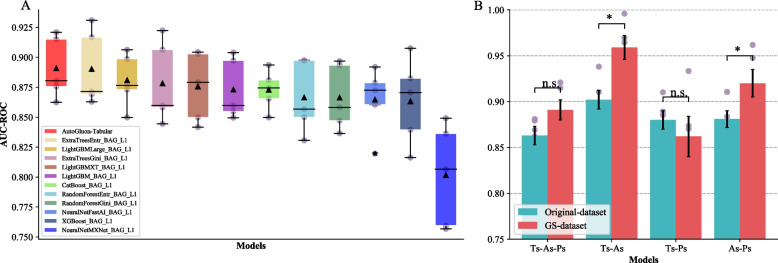
Performance for the models of enzymes synthesizing PSM in *Arabidopsis*. **A** Performance of models built with AutoGluon-Tabular and the algorithms built-in AutoGluon-Tabular. All models were evaluated by mean AUC-ROC from 5 experiments for each model. The black triangles indicate mean values; the black star represents the outliers. **B** Performance of models built with AutoGluon-Tabular with the gold standard (GS) dataset and original dataset for three-classification tasks (terpenoids-alkaloids-phenolics: Ts-As-Ps) and binary-classification tasks (terpenoids-alkaloids: Ts-As; terpenoids-phenolics: Ts-Ps; alkaloids-phenolics: As-Ps). Error bar indicated standard error (SE) for the 5 resamples (see Methods). “*” indicates *P* values less than 0.05 and “n.s.” represents no significant difference

To examine whether the integrating algorithm outperforms all the algorithms implemented in AutoGluon-Tabular, we built three-classification models using GS dataset with AutoGluon-Tabular and all the implemented algorithms (Fig. 2A). 80% genes from the GS dataset were used to train three-classification models, and the remaining 20% genes were utilized as a test set to evaluate the model performance. For each model, five random splits of dataset were repeated. The performance of the model was assessed using the average area under the receiver operating characteristic curve (AUC-ROC), accuracy (ACC) and average F1 score weighted by support (F1; the harmonic mean of precision and recall), and the evaluation scores in five repeats of the three approaches were plotted in Additional file 1: Figure S3 and S6. We observed that the final ensemble model of AutoGluon-Tabular yielded an average AUC-ROC of 0.891 (average ACC = 0.779, average F1 = 0.77), higher than all the built-in conventional ML algorithms (Fig. 2A, Additional file 1: Figure S1 and Table S2). Models built with two deep learning (DL) algorithms (NeuralNetMXNet and NeuralNetFastAI) exhibited the worst performance (*P* < 0.05 from Student’s *t* test), which may result from the relatively small sample size of our data. Thus, in the following analysis, we would only report performance of models built with the ensemble model of AutoGluon-Tabular.

To build predictive models for the enzymes synthesizing PSM, 80% genes from two datasets, the original and GS dataset, were used to train three-classification models with AutoGluon-Tabular (Fig. 2B). The mean AUC-ROC scores for models built with original and GS datasets were 0.863 and 0.891 (original dataset: ACC = 0.711, F1 = 0.706; GS dataset: ACC = 0.765, F1 = 0.756), respectively (Fig. 2B; Additional file 1: Figure S2 and Table S3). The slight AUC-ROC difference (0.028, *P* = 0.105 from Mann-Whitney U test) between these two datasets indicates that the model’s performance was not affected by the reduction of genes for three-classification task in the GS dataset, and that models built with experimentally validated genes only showed minor, if any, advantage over models when computationally annotated genes were included. In addition, we constructed binary-classification models (terpenoids and alkaloids: Ts-As; terpenoids and phenolics: Ts-Ps; alkaloids and phenolics: As-Ps) for any two of the three metabolic gene classes separately (Fig. 2B; Additional file 1: Figure S2 and Table S3). For example, the Ts-Ps means genes synthesizing terpenoids were treated as positive class (first class) and genes synthesizing phenolics were treated as negative class (second class). We found that the models built with the GS dataset significantly outperformed the models built with the original dataset when distinguishing terpenoids and alkaloids (*P* = 1.07e-2 from Mann-Whitney U test), phenolics (P = 3e-2 from Mann-Whitney U test). However, no significant differences in Ts-Ps for the two datasets (Ts-Ps: terpenoids and phenolics, *P* = 0.26 from Mann-Whitney U test). Interestingly, the performance of Ts-Ps models built with GS dataset did not exceed the model built with original dataset, which is contrary to the three and the other two binary-classification models. Hereafter we will focus on the three-classification models using GS dataset for simplicity.

### Screening of important features

**Fig. 3:**
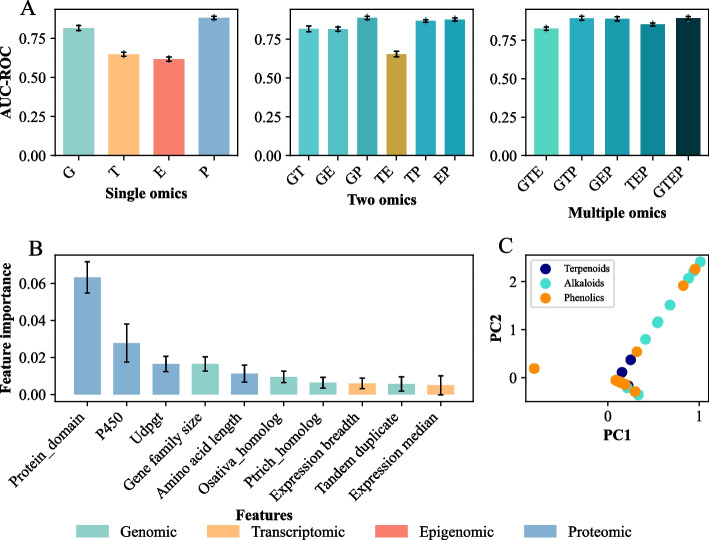
Feature importance and performance of the multi-omics model in *Arabidopsis.* **A** Performance of models built with single, two and multiple omics features (e.g., GTEP: genomic [G], transcriptomic [T], epigenomic [E], and proteomic [P]). Error bar indicates standard errors among 5 replicate runs (see Methods). **B** The top ten important features from the model of three-classification tasks (5 experiments for each model). **C** Principal component analysis (PCA) of Pfam domains for genes involved in the metabolic pathways of terpenoids (purple), alkaloids (turquoise) and phenolics (orange). Scatterplots show the scores of the first two principal components estimated with the feature “protein domains” in *Arabidopsis*

To understand features from which omics data tend to contribute more to the model performance, we classified the features of the *Arabidopsis* GS dataset into four types: genomic (G) [34, 35], transcriptomic (T) [36, 37], epigenomic (E) [38], and proteomic (P) features [39] (Additional file 2: Data S4-7). Features that do not belong to any of the above four categories were excluded from the analyses. Next, we compared the performance of AutoGluon-Tabular models built with different combinations of omics features (Fig. 3A). Specifically, the models were built with all the four types of omics features (GTEP), combinations of three types of omics features (GTE, GTP, GEP, and TEP), combinations of two omics features (GT, GE, GP, TE, TP, and EP) and single-omics features (G, T, E, and P). We found that the model trained with proteomic features had the highest performance (mean AUC-ROC = 0.881, ACC = 0.76, F1 = 0.755), followed by the models trained with genomic (mean AUC-ROC = 0.809, ACC = 0.679, F1 = 0.668), transcriptomic (mean AUC-ROC = 0.648, ACC = 0.475, F1 = 0.462) and epigenomic (mean AUC-ROC = 0.617, ACC = 0.447, F1 = 0.4) features (Fig. 3A; Additional file 1: Figure S3 and Table S4). These results indicate that the proteomic and genomic features might be more informative for predicting enzymes synthesizing PSM than the other two types of features. Surprisingly, we found no improvement in model performance when additional omics features were included to train the models, even for models integrating proteomic and genomic features (GP, mean AUC-ROC: 0.881). These results further suggest that the proteomic and genomic features might be sufficient to distinguish genes involved in metabolic pathways for terpenoids, alkaloids, and phenolics biosynthesis.

Moreover, to identify what features contribute more to the model performance, we examined the mean feature importance from three-classification models with the GS dataset in *Arabidopsis*. A feature score of 0.01 from the AutoGluon-Tabular algorithm indicates a predictive performance drop of 0.01 when this feature’s values are randomly shuffled across instances, and thus a higher feature score suggests more contribution to model performance. Ten features with the highest average feature importance scores were shown in Fig. 3B. The protein domain-related features (Pfam domains were mapped to each gene with a significant match) was the most influential feature (average importance score = 0.063) for enzymes synthesizing PSM prediction, followed by P450 and UDPGT defining whether a gene contains a P450 or UDPGT Pfam domain. Since these top three most important features are all protein domain-related features, we questioned whether genes involved in metabolic pathways of terpenoids, alkaloids and phenolics can be distinguished with protein domain-related features only. Principal component analysis (PCA), an unsupervised learning algorithm, was utilized to assess the utility of the protein domain-related features for enzymes synthesizing PSM prediction (Fig. 3C). Surprisingly, protein domain-related features can’t distinguish genes involved in metabolic pathways of terpenoids, alkaloids and phenolics very well, which may be owing to the lack of Pfam domains or the shared protein domain of some genes. Then another two features related to protein (number of domains and amino acid length) were added in PCA (Additional file 1: Figure S4), which did not improve the prediction of enzymes synthesizing PSM, indicating that a combination of multi-omics features can potentially improve the accurate of enzymes synthesizing PSM predictions.

Besides protein domain-related features, the features related with gene sequences were also important for predicting enzymes synthesizing PSM, such as gene family size, homolog presence (presence of *Arabidopsis* homologs in given species), and presence of tandem duplication (with paralogs within a distance of 10 genes and 100kb) in the study of Moore *et al.* (2019). However, the feature of gene family size was also not able to distinguish three metabolic domains separately as most PSM genes exhibited small gene family size (Additional file 1: Figure S5). Among the top ten important features, there are two expression-related features with relatively lower importance, namely, expression breadth (number of development conditions called with an expression intensity threshold of ≥ 4) and expression median (median expression level in development dataset). In summary, the top ten features were dominated by genomic and proteomic features, which indicates combinations of these essential features may provide sufficient information for enzymes synthesizing PSM prediction. In addition to evaluating the model built with all features (AUC: 0.891 ± 0.026), we further explored the potential benefits of feature selection. We computed feature importances for each omics data type (Additional file 2: Data S8-S11). Features with positive importance scores were considered potentially informative. A model built using a combined set of these positive features achieved an AUC of 0.905 ± 0.025. While the improvement compared to the model using all features is modest, it suggests that feature selection techniques could be a valuable strategy for further optimization, especially when additional data becomes available. Furthermore, the proteomic and genomic features can be captured more readily for non-model species than features from other omics. To evaluate the contribution of these omics data to the model, we built and evaluated models with features from solely genomic and proteomic data, respectively, as well as from both omics data. Interestingly, while a model built using only proteomic features achieved good performance (AUC: 0.863 ± 0.022), incorporating features from both omics data led to a further improvement of model accuracy (AUC: 0.885 ± 0.015) (Additional file 1: Table S5). These results suggest a multi-omics approach can provide additional benefits for prediction tasks. Thus, in the subsequent section, we built cross-species models using features from the two omics data.

### Performance of trans-species prediction

Since the proteomic and genomic features are most informative for predicting enzymes synthesizing PSM, we then asked whether models built with features from both omics in one species can be used to predict enzymes synthesizing PSM in other species. 467 and 544 enzymes synthesizing PSM with experimental and literature support were downloaded for tomato and maize from Plant Metabolic Network (PMN) (https://www.plantcyc.org/), respectively. Considering the fact that the annotations for protein domain and genomic features are continuously updated, and for the sake of the unification of features among three species, we re-extracted features of each gene. Thus, based on the genomic assembly and annotation information, we re-extracted the *Arabidopsis* features to obtain three proteomic related features: amino acid sequence, amino acid sequence length and protein domain, which was similar to proteomic features that were used to build multi-omics models, and two genomic features (i.e., gene length and gene family size). The other genomic features, such as gene sequence and 120 frequencies of bi-nucleotides (see Methods), were discarded due to the lower feature importance. The same features were also extracted for genes in tomato and maize, respectively.

**Fig. 4:**
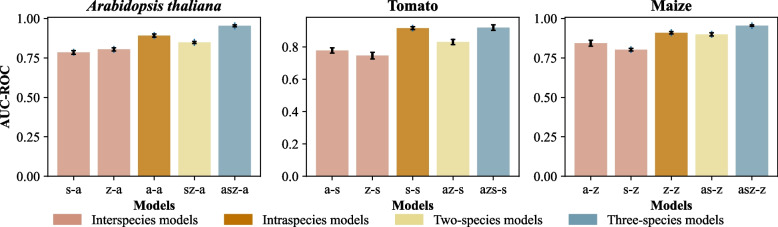
Cross-species validation of enzymes synthesizing PSM. Validation of enzymes synthesizing PSM in *Arabidopsis*, tomato and maize using models trained with genes from other species (the interspecies models, pink), the same species (the intraspecies models, orange), or multiple species (yellow and blue). Model performance was assessed using mean AUC-ROC values. Error bars indicate standard errors among 5 replicate runs (see Methods). Abbreviations: a, *Arabidopsis*; s, tomato; z, maize, asz-a, models trained using genes in the training sets from *Arabidopsis*, tomato and maize were used to predict genes in the *Arabidopsis* test set

Models built using the five genomic and proteomic features of training set genes in single, double or triple species were applied to the test genes in *Arabidopsis* (a for short), tomato (s) and maize (z), separately (Fig. 4). The model performance across species (or interspecies, i.e., model was trained using all GS genes in one species and was evaluated using test genes from another species, e.g., s-a: mean AUC-ROC = 0.785, ACC = 0.588, F1 = 0.559) was lower than the performance of within-species predictions (or intraspecies, i.e., model built with training genes in one species was evaluated with test genes in the same species, e.g., a-a: mean AUC-ROC = 0.924, ACC = 0.765, F1 = 0.756; *P* < 0.001 from Student’s *t* test). Although still worse than intraspecies predictions, the performance of two-species based interspecies models (i.e., model built with all GS genes from two species was evaluated with test genes from another species) was significantly improved compared with that of the single-species based interspecies model (Fig. 4; Additional file 1: Figure S6 and Table S6).

Based on the previous finding that ML models incorporated with training data from multiple species can improve model performance [35], we trained a three-species AutoGluon-Tabular model with *Arabidopsis*, tomato and maize GS datasets, using re-extracted features. We found the validation data of three-species model rendered the best ACC of 0.844. Additionally, we evaluated the performance of our three-species model on an external grape dataset and compare it with seven basic models. While the AutoGluon-Tabular performed better on ACC (0.844) than RF (0.834), LightGBM (0.835), ExtraTrees (0.829), CatBoost (0.827), XGBoost (0.836), NeuralNetFastAI (0.837) and NeuralNetMXNet (0.783) on the validation data, it yielded the lowest ACC (0.603) on the grape dataset. Notably, LightGBM achieved the highest ACC of 0.63 on the grape dataset (Additional file 2: Data S12). Furthermore, to evaluate three-species model in non-model species, such as medicinal plants, the SM genes of *Papaver somniferum* were collected base on the published literatures and KEGG PATHWAY (https://www.genome.jp/kegg/pathway.html) including 22 terpenoids, 13 alkaloids and 38 phenolics genes involved in terpenoid alkaloid, and phenolic biosynthesis respectively. For the genes of *Papaver somniferum*, the performance of AutoGluon-Tabular ensemble model was not as good as that in model species, but still acceptable (AUC-ROC = 0.584, ACC = 0.479, F1 = 0.478). Interestingly, the DL algorithms NeuralNetFastAI gained the best performance (AUC-ROC = 0.69, ACC = 0.589, F1 = 0.607) (Additional file 2: Data S13), which suggested the DL algorithm performed better than ensemble model for three-classification task when the training gene set was increased to over 1000 genes (Additional file 2: Data S3). Further scrutiny showed that the main failure lied in the distinction between alkaloids and phenolics (6 alkaloids predicted to phenolics; 11 phenolics predicted to alkaloids). For example, as for the alkaloid gene “113340172”, the predicted probability for alkaloids is 0.39, for phenolics is 0.52 and for terpenoids is 0.09. Two factors are possibly accountable for the decrease of performance: (1) the distinct source of training (exclusively from PMN) and test dataset; (2) the limited representation of the full diversity of metabolic pathways within each of the tree main PSM categories. Taken together, it implied that more samples or features are needed for a better prediction of unknown gene in non-model species.

Finally, three-species models, which were trained with all GS genes from all the three species and evaluated with test genes in the species in question, exhibited either equivalent or superior performance to intraspecies predictions (Fig. 4).

## Discussion

Researchers have explored the application of ML methods in prediction of genes synthesizing secondary metabolites (SM) versus primary metabolites (PM) [26] and participating in specific secondary metabolite pathways [20]. Models distinguishing SM genes from PM genes had great performance (accuracy = 86%), while models for individual pathways failed in predicting genes to correct pathways (accuracy = 58.3% for benchmark genes). The failure can be due to several reasons: 1) the training gene sets within individual pathways had limited sizes; 2) that work was done in tomato where only few experimentally validated metabolic pathway genes are available; 3) only transcriptome data was used in that study. Considering these aspects and the trade-off between the refinement and accuracy of gene prediction, we established models distinguishing genes participating in the biosynthesis of three main categories of secondary metabolites (terpenoids, alkaloids, and phenolics) in *Arabidopsis* using the same features as in Moore *et al*. (2019). Our prediction model demonstrated a mean accuracy of 83.2% and the best accuracy of 86.6% at predicting genes participating in a broader classification of secondary metabolites. In addition, we came up with a minimized set of features with no decreases in prediction performance, which pronounced broader applications of our models especially in non-model species. The reliable application of *Arabidopsis* model in cultivated tomato, maize, grape and *Papaver somniferum* further supports this.

The upper bound of the model is determined by the dataset and features [40]. A dataset with accurate labels and balanced number of items under each label was crucial for a reasonable model. In this study, we selected genes with experimental validation as the GS dataset and validated the reliability of the gold standard dataset by comparing the model performance with the original dataset. The GS dataset contained a total of 594 genes, which was comparable to the number of specialized metabolic genes (649) utilized in Moore et al. (2019). An automated ML algorithm was employed to build models, because the algorithm neither necessitates a large amount of data like DL algorithms nor requires manual tuning of parameters like conventional ML algorithms [41, 42]. We found in our GS dataset, the model AutoGluon-Tabular outperformed models using individual DL algorithms implemented in AutoGluon-Tabular (Fig. 2A), which usually require a large amount of data for the training and did not work well with such a small amount of data. For example, Ma et al. [43] utilized 10,321 antimicrobial peptides (AMPs) and 3,030,124 Non-AMP to build and optimize DL models for identifying AMPs from the human gut microbiome. Chen et al. [44] employed 30 million sequences (4-kb windows with 100-base pair step size) in the human genome to build a robust DL model “Sei” for discovering the regulatory basis of traits and diseases. In contrast, our model might be applicable in specialized tasks with limited amounts of data, such as human diseases or plant metabolic pathways [45, 46].

Among the features we investigated, genomics and proteomics related features played a significant role in predicting genes synthesizing the three categories of PSM in our study. Importantly, these features have also been shown to play a pivotal role in gene prediction in other studies. For example, gene family size and specialized protein domain (P450) provided significant information in models predicting the primary and specialized metabolism genes constructed by Moore et al. (2019). In addition, the sequence features of genes, which can be calculated solely from genomic sequences and gene structural annotation, contributed significantly to the prediction of genes that transcriptionally responded to cold abiotic stress [35]. In conclusion, the genomics and proteomics related features enabled us to build models that outperformed those constructed with all multi-omics features. In addition, due to the relatively easy extraction of these features from species with genome assemblies, our practice can overcome the limitation of adaptability of models when applied across non-model species, especially for medicinal plants. Even though a small feature set (five key features) facilitated interpretability and model performance, it inherently restricts the model's ability to learn and predict unknown enzymes synthesizing PSMs due to limited knowledge. To address this limitation and enhance the model's capacity, future investigations could focus on exploring the integration of pre-trained protein language models and incorporating features specifically tailored to different plant species or specific secondary metabolite classes (e.g., alkaloids and phenolics).

Using only five genomic and proteomic features, the predicting models rendered an average AUC-ROC of > 0.8 across three species, which is comparable to the cross-species model performance for predicting cold responsive genes in Poaceae [35]. Furthermore, the successful prediction of SM genes in grape and *Papaver somniferum* suggests the potential of our models for identifying SM genes synthesizing the surveyed three categories of PSM among genes with unknown function based on data in non-model species. However, further validation on a broader range of non-model species is warranted to evaluate the models’ generalizability. Our cross-species validation was based on four species from three different families spanning monocots and eudicots. Despite the distant evolutionary relationships among these species, the high accuracy across species indicated that our models have great application potential for predicting genes participating in specialized metabolic pathways in some non-model plant species. For example, in traditional medicinal plants, which are a rich source of highly diverse specialized metabolites with critical pharmacological properties [16], our models can be potentially used to identify unknown genes synthesizing effective compounds. In the future, the prediction accuracy of enzymes synthesizing PSM will be further improved along with the explosion of plant omics data and the increasing accumulation of gene annotation information. Our study manifested the great potential of machine learning models in unearthing the biosynthesis pathway of effective biochemical molecules in plant species.

## Conclusions

Here, we constructed a GS dataset with multi-omics features for genes synthesizing three specialized metabolites: terpenoids, alkaloids and phenolics, in the model species *Arabidopsis*. A three-classification model was built using this dataset and the ML algorithm AutoGluon-Tabular, rendering a mean AUC-ROC of 0.891 (Fig. 2A). Among the features we investigated, genomic and proteomic features were selected as the crucial omics features for predicting genes synthesizing the three categories of PSM (Fig. 3). The key features that were re-extracted for genes in *Arabidopsis*, tomato and maize were utilized to build new models (Fig. 4), which exhibited high prediction accuracy across species. Our results illustrate the application potential of models built in species with relatively abundant experimentally validated pathway annotations to data-poor species. In addition, our model is also beneficial for predicting unknown enzymes synthesizing PSM, and may guide experimental design and save significant cost [47, 48].

## Methods

### Datasets

For training models, we downloaded specialized metabolic genes and pathway annotation in three species (namely, *Arabidopsis* [*Arabidopsis thaliana col-0*], tomato [*Solanum lycopersicum*], and maize [*Zea mays B73*]) from Plant Metabolic Network (PMN 15.5) (https://www.plantcyc.org/). Datasets from these three species were used in this study owing to the high data quality [49] and comprehensive pathway annotation information. Based on the metabolic domains of PMN, a label (either terpenoids, alkaloids, or phenolics) was obtained for each gene. Genes assigned to ≥2 domains were not included. In total, we identified 951 *Arabidopsis* genes, 1234 tomato genes, and 1179 maize genes. Furthermore, to obtain gold standard (GS) gene sets, genes without experimental and literature support (have a PubMed ID) were excluded, resulting in 594 *Arabidopsis* genes, 467 tomato genes, and 544 maize genes (Additional file 2: Data S2).

For additional validation of the model, we collected two different datasets. The first dataset consisted of 1083 grape SM genes that were collected from PMN with the same data processing as *Arabidopsis*. This dataset included 285 genes involved in terpene biosynthesis, 313 genes involved in alkaloid biosynthesis, and 485 genes involved in phenolic biosynthesis (Additional file 2: Data S12). The second dataset was composed of 73 poppy SM genes from Kyoto Encyclopedia of Genes and Genomes (KEGG; https://www.genome.jp/kegg/) including 22 terpenoids (11 sesquiterpenoid and triterpenoid biosynthesis and 11 diterpenoid biosynthesis genes), 13 alkaloids (13 isoquinoline alkaloid biosynthesis genes) and 38 phenolics (8 anthocyanin biosynthesis and 30 coumarin biosynthesis genes) genes (Additional file 2: Data S13).

### Feature acquirement

For analyzing feature importance and building models in various species, two feature sets were constructed on the GS dataset of the model species *Arabidopsis*.

First, we collected a comprehensive set of features, including four categories (e.g., genomic sequence information, expression/co-expression, chromatin accessibility, protein domain) [26]. The features of the protein domain were transformed from one-hot coding to text string to regain information about the domain. Two approaches were utilized to process sequence features. One approach was to maintain the raw information of the sequence by splitting each nucleotide with space and treating it as one feature. The other was to calculate frequencies of all individual nucleotides (4 features) and dinucleotides (16 features) for each of six sequence regions: the CDS, intron, estimated 5’ UTR, estimated 3’ UTR, 1 kb upstream of the 5’ UTR starting site, and 1 kb downstream of the 3’ UTR ending site, which resulted in 120 features as described in Meng et al. (2021). Overall, 234 features were scored and collected for each gene (Additional file 2: Data S1).

Second, to expand the model application, five important features related to genomics and proteomics were selected and re-extracted: protein domain, amino acid sequence, amino acid sequence length, gene length and gene family size. The protein domain(s) of each gene was obtained from Pfam v35.0-A using amino acid sequence with sequence-searching software HMMER (3.1b1; http://hmmer.org/) (hmmscan -o out.txt -E 0.001 --cpu 2 ./Pfam-A.hmm input_file) [50]. Amino acid sequence, amino acid sequence length, and gene length were obtained based on gene names and genome assemblies with Bioawk (https://github.com/lh3/bioawk). Genome assemblies, gene annotation and protein sequences of genes in *Arabidopsis* (TAIR10), tomato (SL3.0), maize (Zm-B73-REFERENCE-NAM-4.0) and grape (*Vitis vinifera*) were downloaded from Ensemble Genomes [51]. The feature gene family size was obtained from a previous work [49]. Genes with unknown sequence or protein domain were excluded in the analysis. Summaries of feature values for *Arabidopsis*, tomato, and maize were deposited in Additional file 2: Data S2 and for grape in Additional file 2: Data S12. The poppy SM genes were extracted from KEGG with a python script and their features were further collected (Additional file 2: Data S13).

### AutoGluon-Tabular model training, classification, and evaluation

Two strategies were taken to generate holdout test data: one for within-species prediction; the other for cross-species prediction. For within-species prediction, all genes were partitioned into training (80%) and test (20%) subsets. For cross-species predictions, models were trained with all the GS genes from other species and were applied on genes in the test (20%) subset in a given species, in order to ensure that the cross-species model performance is comparable to the within-species performance. Models were trained by using the AutoGluon-Tabular algorithm in the python (v3.8.13) package “autogluon” (v0.4.2) [27]. Compared with conventional ML algorithms, AutoGluon-Tabular can train a ML model with relative high accuracy from an unprocessed tabular dataset such as a CSV file by automatically recognizing the data type in each column, including text data. Meanwhile, it can significantly save considerable time in parameter tuning and feature engineering. In this study, we utilized two hyperparameters. The parameter “num_bag_folds” (the fold number of cross-validation) was set as five (num_bag_folds=5), and the “time_limit” (the training model’s limit time) was set as 1000 (time_limit=1000), which means AutoGluon-Tabular trains multiple ML models and integrates the models to an ensemble model within 1000 seconds. Other hyperparameters, such as the number of folds for model-training vs. validation and the type of prediction problem (binary, multi-class classification or regression), were automatically optimized by the algorithm AutoGluon-Tabular. To evaluate the performance of AutoGluon-Tabular and the models implemented in AutoGluon-Tabular, we used three evaluation statistics: accuracy (ACC), average F1 score weighted by support (F1_weighted), and macro-averaged area under the receiver operating characteristic curve (AUC-ROC) in package ‘scikit-learn’ (https://scikit-learn.org/). We evaluated all the algorithms using five randomly (random_seed = 1 ~ 5) generated training and test splits, and the mean and standard error of the evaluation metrics across these five replicate runs were reported.

### Supplementary Information

**Additional file 1: Figure S1.** Performance in predicting enzymes synthesizing PSM in Arabidopsis. **Figure S2.** Performance in predicting enzymes synthesizing PSM in Arabidopsis for three-classification (terpenoids-alkaloids-phenolics: Ts-As-Ps) and binary-classification (terpenoids-alkaloids: Ts-As; terpenoids-phenolics: Ts-Ps; alkaloids-phenolics: As-Ps; n = 5 experiments for each model). **Figure S3.** Performance of models built with single omics features and multiple omics features (e.g., GTEP: genomic [G], transcriptomic [T], epigenomic [E], and proteomic [P]). **Figure S4.** Principal component analysis (PCA) analysis of protein domain-related features. **Figure S5.** Distribution of genomic-related feature gene family size. **Figure S6.** Cross-species validation of enzymes synthesizing PSM. **Table S1.** Hyperparameter settings for the seven baseline models and AutoGluon-Tabular. **Table S2.** Performance in predicting enzymes synthesizing PSM in Arabidopsis. **Table S3.** Performance of models built with AutoGluon-Tabular with the gold standard (GS) dataset and original dataset. **Table S4.** Performance of models built with single omics features and multiple omics features. **Table S5.** Cross-species prediction of enzymes synthesizing PSM. **Table S6.** Performance of selected features from genomic, proteomic.

**Additional file 2:** **Data S1.** Original data for Arabidopsis thaliana. **Data S2.** The detailed description of features that utilized in this study. **Data S3.** Gold standard data for Arabidopsis thaliana (AT), Solanum lycopersicum (Solyc) and Zea mays B73 (Zm). **Data S4.** Values of each gene for genomic features for Arabidopsis thaliana. **Data S5.** Values of each gene for transcriptomic features for Arabidopsis thaliana. **Data S6.** Values of each gene for epigenomic features for Arabidopsis thaliana. **Data S7.** Values of each gene for proteomic features for Arabidopsis thaliana. **Data S8.** Feature importance for genomic. **Data S9.** Feature importance for transcriptomic. **Data S10.** Feature importance for epigenomic. **Data S11.** Feature importance for proteomic. **Data S12.** Features of each gene and performance in predicting enzymes synthesizing PSM in grape. **Data S13.** Features of each gene and performance in predicting enzymes synthesizing PSM in Papaver somniferum.

## Data Availability

The datasets and codes are available at https://github.com/baiwenhuim/ML-pre-GSPSM.

## Abbreviations

PSM: Plant specialized metabolites
ML: Machine learning
RF: Random Forests
LightGBM: LightGBM boosted trees
CatBoost: CatBoost boosted trees
ExtraTrees: Extremely Randomized Trees
AUC-ROC: Average area under the receiver operating characteristic curve
ACC: Accuracy
DL: Deep learning
Ts: Terpenoids
As: Alkaloids
Ps: Phenolics
GS: Gold standard
SE: Standard error
G: Genomic
T: Transcriptomic
E: Epigenomic
P: Proteomic
PCA: Principal component analysis
PMN: Plant Metabolic Network
AMPs: Antimicrobial peptides
