# Supplementary material for: Machine learning assists prediction of genes responsible for plant specialized metabolite biosynthesis by integrating multi-omics data
Source: BMC Genomics. 2024 Apr 29;25:418. doi: 10.1186/s12864-024-10258-6 (PMC11057162; doi:10.1186/s12864-024-10258-6)
Supplement: Supplementary file 1 — Additional file 1: Figure S1. Performance in predicting enzymes synthesizing PSM in Arabidopsis. Figure S2. Performance in predicting enzymes synthesizing PSM in Arabidopsis for three-classification (terpenoids-alkaloids-phenolics: Ts-As-Ps) and binary-classification (terpenoids-alkaloids: Ts-As; terpenoids-phenolics: Ts-Ps; alkaloids-phenolics: As-Ps; n = 5 experiments for each model). Figure S3. Performance of models built with single omics features and multiple omics features (e.g., GTEP: genomic [G], transcriptomic [T], epigenomic [E], and proteomic [P]). Figure S4. Principal component analysis (PCA) analysis of protein domain-related features. Figure S5. Distribution of genomic-related feature gene family size. Figure S6. Cross-species validation of enzymes synthesizing PSM. Table S1. Hyperparameter settings for the seven baseline models and AutoGluon-Tabular. Table S2. Performance in predicting enzymes synthesizing PSM in Arabidopsis. Table S3. Performance of models built with AutoGluon-Tabular with the gold standard (GS) dataset and original dataset. Table S4. Performance of models built with single omics features and multiple omics features. Table S5. Cross-species prediction of enzymes synthesizing PSM. Table S6. Performance of selected features from genomic, proteomic. [file 12864_2024_10258_MOESM1_ESM.docx]

**Supplemental figure and table legends:**

**Figure S1.** Performance in predicting enzymes synthesizing PSM in Arabidopsis.

**Figure S2.** Performance in predicting enzymes synthesizing PSM in Arabidopsis for three-classification (terpenoids-alkaloids-phenolics: Ts-As-Ps) and binary-classification (terpenoids-alkaloids: Ts-As; terpenoids-phenolics: Ts-Ps; alkaloids-phenolics: As-Ps; n = 5 experiments for each model).

**Figure S3. Performance of models built with single omics features and multiple omics features (e.g., GTEP: genomic [G], transcriptomic [T], epigenomic [E], and proteomic [P]).**

**Figure S4. Principal component analysis (PCA) analysis of protein domain-related features.**

**Figure S5. Distribution of genomic-related feature gene family size.**

**Figure S6. Cross-species validation of enzymes synthesizing PSM.**

**Table S1.** Hyperparameter settings for the seven baseline models and AutoGluon-Tabular.

**Table S2.** Performance in predicting enzymes synthesizing PSM in Arabidopsis.

**Table S3.** Performance of models built with AutoGluon-Tabular with the gold standard (GS) dataset and original dataset.

**Table S4.** Performance of models built with single omics features and multiple omics features.

**Table S5.** Performance of selected features from genomic, proteomic.

**Table S6.** Performance of cross-species prediction.

**Supplemental Figures:**


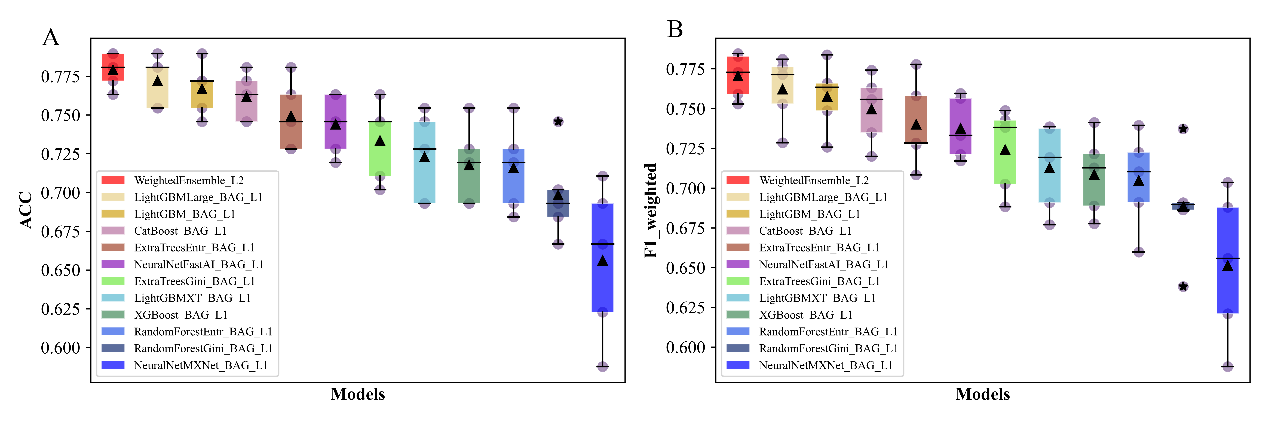


**Figure S1.** **Performance in predicting enzymes synthesizing PSM in Arabidopsis** (n = 5 experiments for each model).

1. The accuracy (ACC) achieved by models trained by using the algorithm AutoGluon-Tabular and algorithms build-in it.
2. The F1_weighted achieved by models trained by using the algorithm AutoGluon-Tabular and algorithms build-in it.


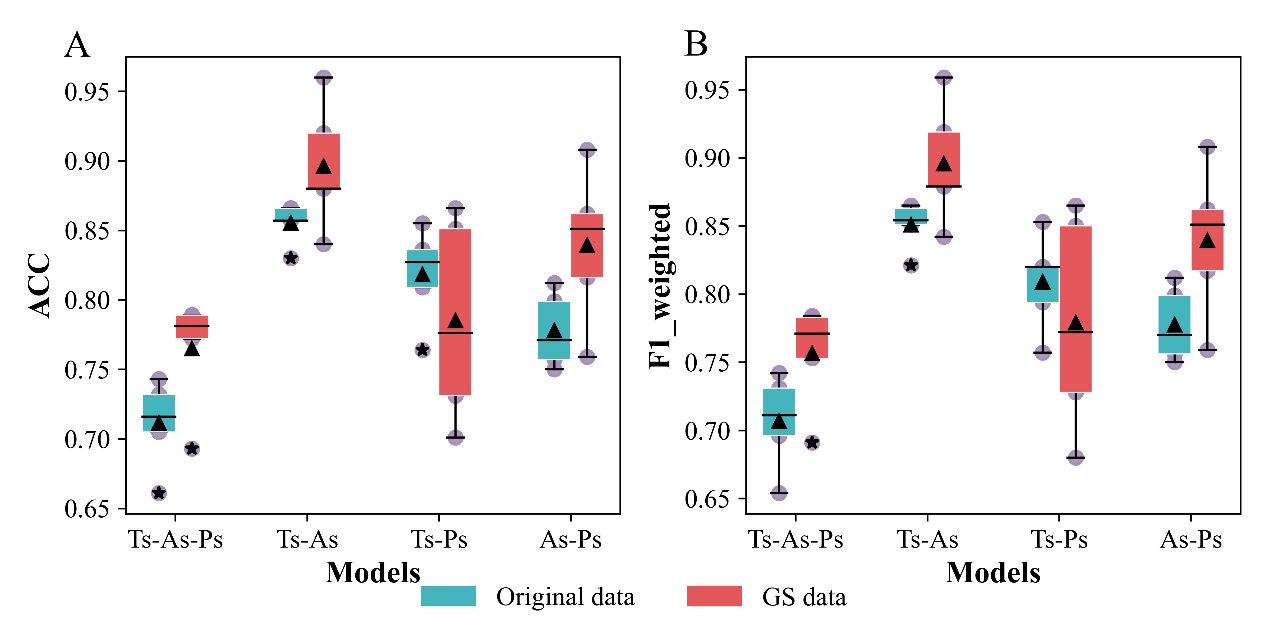


**Figure S2. Performance in predicting enzymes synthesizing PSM in Arabidopsis for three-classification (terpenoids-alkaloids-phenolics: Ts-As-Ps) and binary-classification (terpenoids-alkaloids: Ts-As; terpenoids-phenolics: Ts-Ps; alkaloids-phenolics: As-Ps; n = 5 experiments for each model)**.

1. The accuracy (ACC) achieved by models trained by using the algorithm AutoGluon-Tabular.
2. F1_weighted achieved by models trained by using the algorithm AutoGluon-Tabular.


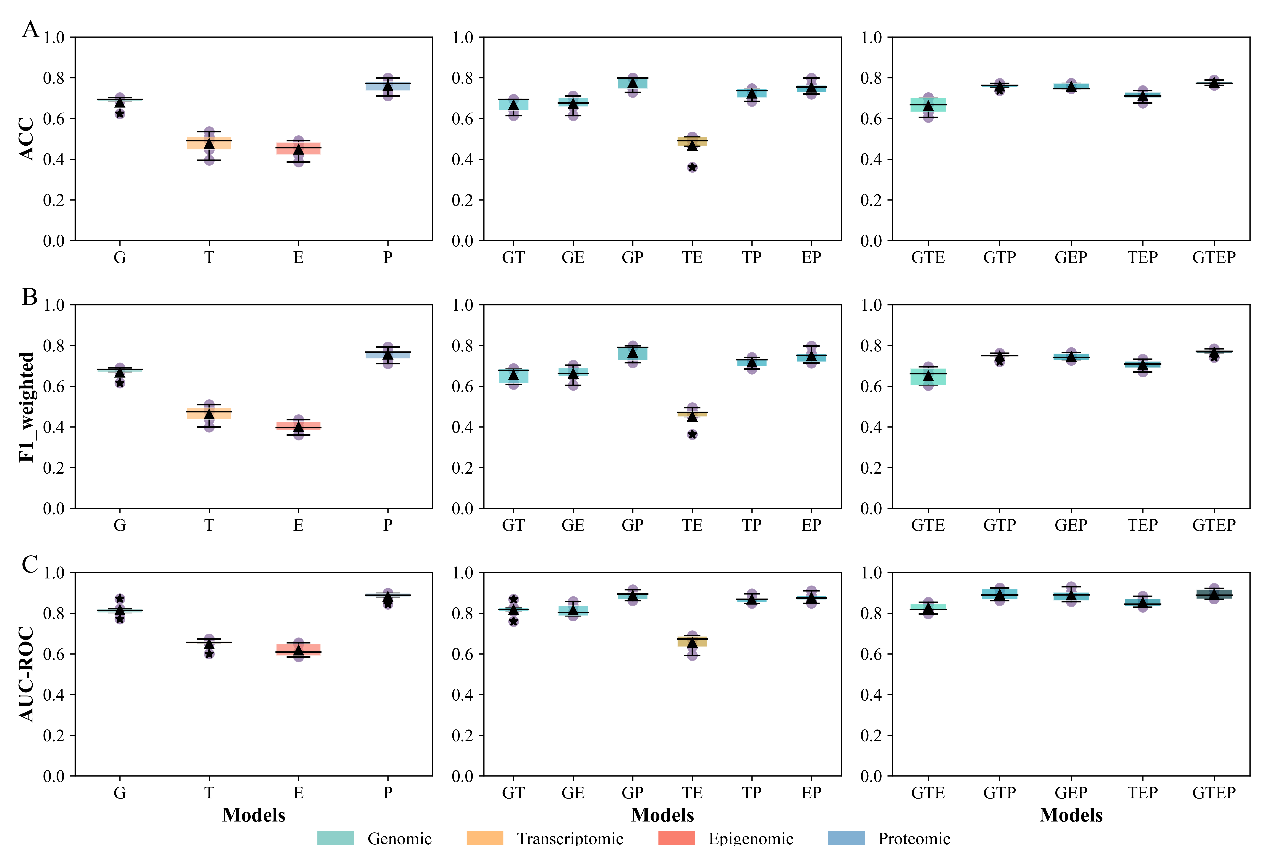


**Figure S3. Performance of models built with single omics features and multiple omics features (e.g., GTEP: genomic [G], transcriptomic [T], epigenomic [E], and proteomic [P]).**

1. The accuracy (ACC) achieved by models trained by using the algorithm AutoGluon-Tabular.
2. F1_weighted achieved by models trained by using the algorithm AutoGluon-Tabular.
3. The mean area under receiver operating characteristic curve (AUC-ROC) achieved by models trained by using the algorithm AutoGluon-Tabular.


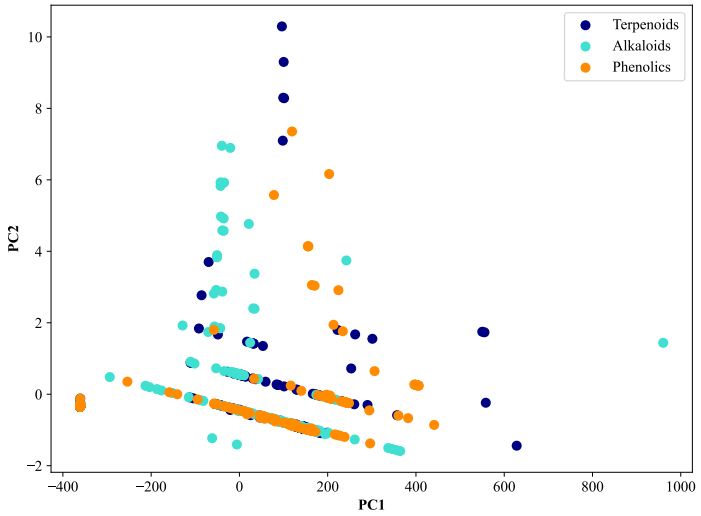


**Figure S4. Principal component analysis (PCA) analysis of protein domain-related features. The features (Pfam domain, number of domains and Amino acid length) for genes involved in the metabolic pathways of terpenoids (purple) alkaloids (white blue) and phenolics (orange).**

**
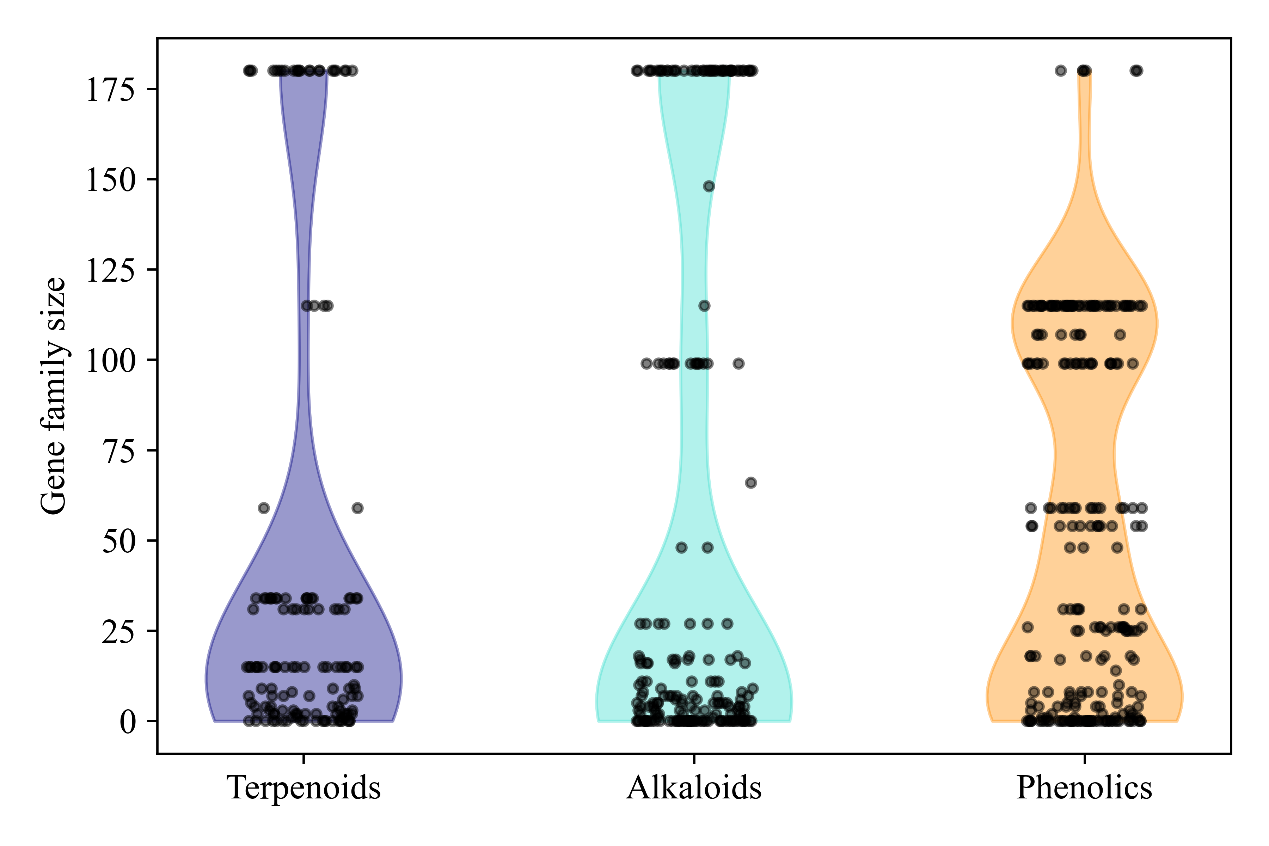
**

**Figure S5. Distribution of genomic-related feature gene family size.** The violin plot of genomic-related feature gene family size in three plant specialized metabolite: terpenoids, alkaloids and phenolics, and the black point represent the value of gene family size.


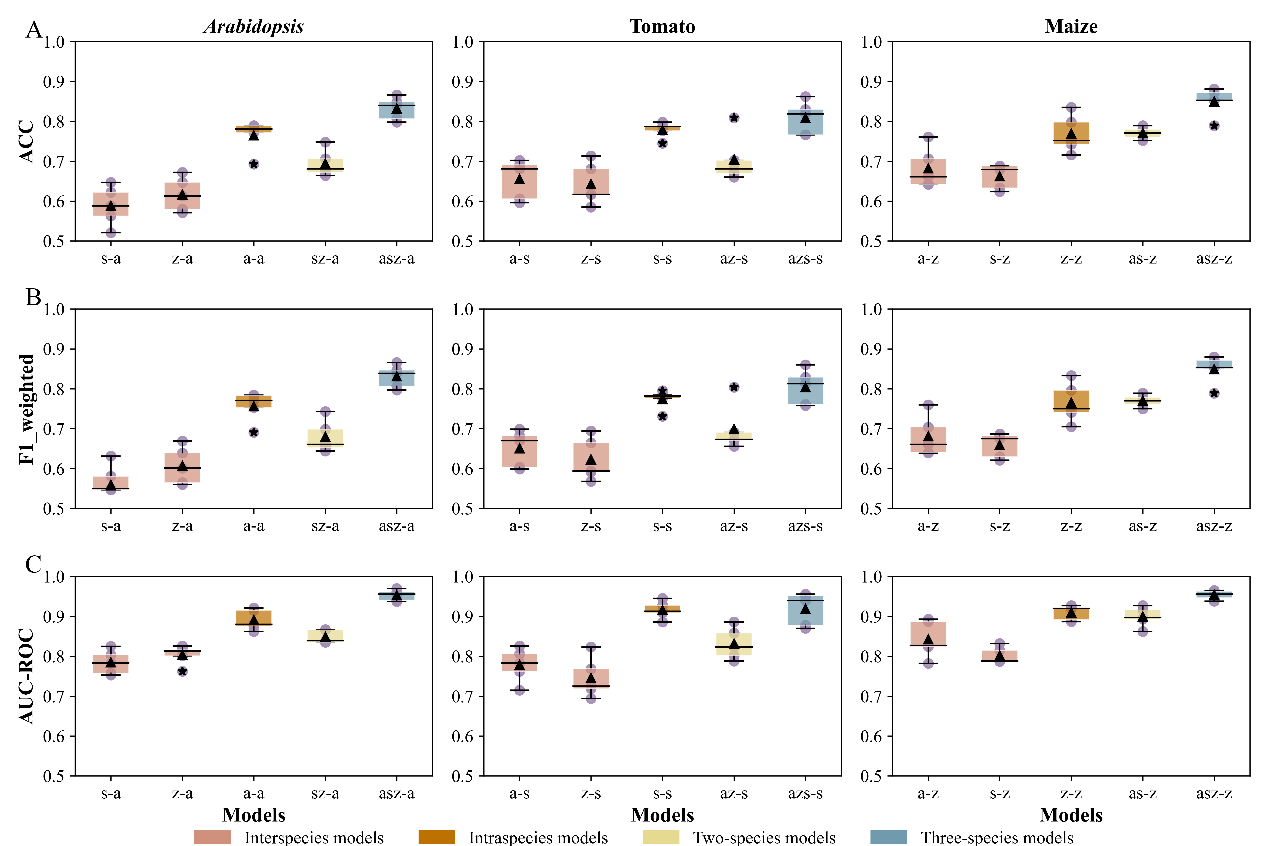


**Figure S6. Cross-species prediction of enzymes synthesizing PSM.**

1. The accuracy (ACC) achieved by models trained by using the algorithm AutoGluon-Tabular in three species (Arabidopsis, tomato and maize).
2. F1_weighted achieved by models trained by using the algorithm AutoGluon-Tabular in three species (Arabidopsis, tomato and maize).
3. The mean AUC-ROC achieved by models trained by using the algorithm AutoGluon-Tabular.

**Supplemental Tables:**

**Table S1. Hyperparameter settings for the seven baseline models and AutoGluon-Tabular.**

| Models | hyperparameters | | | | | |
| --- | --- | --- | --- | --- | --- | --- |
|  | \| use_orig_features \| \| --- \| | max_base_models | max_base_models_per_type | save_bag_folds | use_child_oof | weights |
| RandomForest | TRUE | 25 | 5 | TRUE | TRUE | 0.123077 |
| LightGBM | TRUE | 25 | 5 | TRUE | None | 0.169231 |
| CatBoost | TRUE | 25 | 5 | TRUE | None | 0.292308 |
| ExtraTrees | TRUE | 25 | 5 | TRUE | TRUE | 0.338462 |
| XGBoost | TRUE | 25 | 5 | TRUE | None | 0 |
| NeuralNetFastAI | TRUE | 25 | 5 | TRUE | None | 0.076923 |
| NeuralNetMXNet | TRUE | 25 | 5 | TRUE | None | 0 |
| AutoGluon-Tabular | FALSE | 25 | 5 | TRUE | None | 1 |

**Table S2. Performance in predicting enzymes synthesizing PSM in Arabidopsis.**

| model | accuracy | f1_weighted | roc_auc_ovo_macro |
| --- | --- | --- | --- |
| AutoGluon-Tabular | **0.779 ± 0.011** | **0.770 ± 0.014** | **0.891 ± 0.026** |
| LightGBMLarge_BAG_L1 | 0.772 ± 0.016 | 0.762 ± 0.022 | 0.890 ± 0.031 |
| LightGBM_BAG_L1 | 0.767 ± 0.017 | 0.757 ± 0.022 | 0.881 ± 0.022 |
| CatBoost_BAG_L1 | 0.761 ± 0.016 | 0.750 ± 0.022 | 0.878 ± 0.034 |
| ExtraTreesEntr_BAG_L1 | 0.749 ± 0.023 | 0.740 ± 0.028 | 0.876 ± 0.029 |
| NeuralNetFastAI_BAG_L1 | 0.744 ± 0.020 | 0.737 ± 0.020 | 0.873 ± 0.026 |
| ExtraTreesGini_BAG_L1 | 0.733 ± 0.026 | 0.724 ± 0.027 | 0.873 ± 0.016 |
| LightGBMXT_BAG_L1 | 0.723 ± 0.029 | 0.713 ± 0.028 | 0.866 ± 0.030 |
| XGBoost_BAG_L1 | 0.718 ± 0.026 | 0.708 ± 0.025 | 0.866 ± 0.027 |
| RandomForestEntr_BAG_L1 | 0.716 ± 0.028 | 0.705 ± 0.031 | 0.865 ± 0.028 |
| RandomForestGini_BAG_L1 | 0.698 ± 0.029 | 0.688 ± 0.035 | 0.863 ± 0.036 |
| NeuralNetMXNet_BAG_L1 | 0.656 ± 0.051 | 0.651 ± 0.048 | 0.802 ± 0.043 |

**Table S3. Performance of models built with AutoGluon-Tabular with the gold standard (GS) dataset and original dataset.**

| dataset | tasks | accuracy | f1_weighted | roc_auc_ovo_macro |
| --- | --- | --- | --- | --- |
| GS | T-A-P | 0.765 ± 0.041 | 0.756 ± 0.039 | 0.891 ± 0.026 |
|  | T-A | 0.896 ± 0.046 | 0.896 ± 0.045 | 0.959 ± 0.028 |
|  | T-P | 0.785 ± 0.072 | 0.779 ± 0.079 | 0.862 ± 0.048 |
|  | A-P | 0.839 ± 0.056 | 0.839 ± 0.055 | 0.920 ± 0.033 |
| Original | T-A-P | 0.711 ± 0.032 | 0.707 ± 0.034 | 0.863 ± 0.023 |
|  | T-A | 0.855 ± 0.015 | 0.851 ± 0.018 | 0.902 ± 0.023 |
|  | T-P | 0.818 ± 0.035 | 0.809 ± 0.036 | 0.880 ± 0.022 |
|  | A-P | 0.778 ± 0.027 | 0.777 ± 0.027 | 0.881 ± 0.020 |

**Table S4. Performance of models built with single omics features and multiple omics features.**

|  | omics | accuracy | f1_weighted | roc_auc_ovo_macro |
| --- | --- | --- | --- | --- |
| single | G | 0.679 ± 0.032 | 0.668 ± 0.030 | 0.816 ± 0.036 |
|  | T | 0.475 ± 0.055 | 0.462 ± 0.044 | 0.648 ± 0.028 |
|  | E | 0.447 ± 0.044 | 0.400 ± 0.031 | 0.617 ± 0.032 |
|  | P | 0.760 ± 0.035 | 0.755 ± 0.032 | 0.881 ± 0.022 |
| dual | GT | 0.667 ± 0.037 | 0.654 ± 0.040 | 0.816 ± 0.039 |
|  | GE | 0.672 ± 0.039 | 0.662 ± 0.038 | 0.814 ± 0.031 |
|  | GP | 0.774 ± 0.034 | 0.764 ± 0.040 | 0.887 ± 0.023 |
|  | TE | 0.467 ± 0.062 | 0.450 ± 0.052 | 0.655 ± 0.041 |
|  | TP | 0.723 ± 0.028 | 0.717 ± 0.025 | 0.867 ± 0.018 |
|  | EP | 0.752 ± 0.031 | 0.748 ± 0.034 | 0.877 ± 0.023 |
| three | GTE | 0.662 ± 0.043 | 0.650 ± 0.044 | 0.825 ± 0.023 |
|  | GTP | 0.758 ± 0.013 | 0.747 ± 0.016 | 0.892 ± 0.029 |
|  | GEP | 0.756 ± 0.014 | 0.744 ± 0.018 | 0.888 ± 0.030 |
|  | TEP | 0.711 ± 0.024 | 0.705 ± 0.025 | 0.852 ± 0.023 |
| four | GTEP | 0.775 ± 0.010 | 0.766 ± 0.016 | 0.893 ± 0.025 |

Note: Performance of models built with single omics features and multiple omics features. (e.g., GTEP: genomic [G], transcriptomic [T], epigenomic [E], and proteomic [P]). (as shown in Figure S3). **Table S5. Performance of selected features from genomic, proteomic.**

|  | accuracy | f1_weighted | roc_auc_ovo_macro |
| --- | --- | --- | --- |
| genomic | 0.632 ± 0.031 | 0.626 ± 0.026 | 0.781 ± 0.008 |
| proteomic | 0.756 ± 0.028 | 0.750 ± 0.028 | 0.863 ± 0.022 |
| genomic+ proteomic | 0.784 ± 0.025 | 0.780 ± 0.025 | 0.885 ± 0.015 |

**Table S6. Performance of cross-species prediction.**

| species | model | accuracy | f1_weighted | roc_auc_ovo_macro |
| --- | --- | --- | --- | --- |
| *Arabidopsis* | s-a | 0.588 ± 0.049 | 0.559 ± 0.053 | 0.785 ± 0.030 |
|  | z-a | 0.617 ± 0.043 | 0.607 ± 0.047 | 0.804 ± 0.025 |
|  | a-a | 0.765 ± 0.041 | 0.756 ± 0.039 | 0.891 ± 0.026 |
|  | sz-a | 0.694 ± 0.034 | 0.680 ± 0.041 | 0.848 ± 0.017 |
|  | asz-a | 0.832 ± 0.029 | 0.831 ± 0.029 | 0.953 ± 0.014 |
| tomato | a-s | 0.655 ± 0.050 | 0.651 ± 0.046 | 0.778 ± 0.043 |
|  | z-s | 0.643 ± 0.053 | 0.623 ± 0.054 | 0.746 ± 0.051 |
|  | s-s | 0.779 ± 0.020 | 0.774 ± 0.025 | 0.916 ± 0.022 |
|  | az-s | 0.704 ± 0.061 | 0.699 ± 0.060 | 0.831 ± 0.040 |
|  | azs-s | 0.809 ± 0.042 | 0.804 ± 0.044 | 0.919 ± 0.042 |
| maize | a-z | 0.682 ± 0.051 | 0.681 ± 0.051 | 0.843 ± 0.047 |
|  | s-z | 0.662 ± 0.031 | 0.659 ± 0.031 | 0.802 ± 0.021 |
|  | z-z | 0.769 ± 0.047 | 0.765 ± 0.050 | 0.909 ± 0.018 |
|  | as-z | 0.771 ± 0.015 | 0.770 ± 0.015 | 0.899 ± 0.025 |
|  | asz-z | 0.850 ± 0.036 | 0.849 ± 0.036 | 0.954 ± 0.011 |

Note: Cross-species prediction of enzymes synthesizing PSM (as shown in Figure S6).
